# Non-Markovian full counting statistics in quantum dot molecules

**DOI:** 10.1038/srep08978

**Published:** 2015-03-10

**Authors:** Hai-Bin Xue, Hu-Jun Jiao, Jiu-Qing Liang, Wu-Ming Liu

**Affiliations:** 1 College of Physics and Optoelectronics, Taiyuan University of Technology, Taiyuan 030024, China; 2 Department of Physics, Shanxi University, Taiyuan 030006, China; 3 Institute of Theoretical Physics, Shanxi University, Taiyuan 030006, China; 4 Beijing National Laboratory for Condensed Matter Physics, Institute of Physics, Chinese Academy of Sciences, Beijing 100190, China

## Abstract

Full counting statistics of electron transport is a powerful diagnostic tool for probing the nature of quantum transport beyond what is obtainable from the average current or conductance measurement alone. In particular, the non-Markovian dynamics of quantum dot molecule plays an important role in the nonequilibrium electron tunneling processes. It is thus necessary to understand the non-Markovian full counting statistics in a quantum dot molecule. Here we study the non-Markovian full counting statistics in two typical quantum dot molecules, namely, serially coupled and side-coupled double quantum dots with high quantum coherence in a certain parameter regime. We demonstrate that the non-Markovian effect manifests itself through the quantum coherence of the quantum dot molecule system, and has a significant impact on the full counting statistics in the high quantum-coherent quantum dot molecule system, which depends on the coupling of the quantum dot molecule system with the source and drain electrodes. The results indicated that the influence of the non-Markovian effect on the full counting statistics of electron transport, which should be considered in a high quantum-coherent quantum dot molecule system, can provide a better understanding of electron transport through quantum dot molecules.

Full counting statistics^1^ (FCS) of electron transport through mesoscopic system has attracted considerable attention both experimentally and theoretically because it can provide a deeper insight into the nature of electron transport mechanisms, which cannot be obtained from the average current^2^,^3^,^4^,^5^,^6^,^7^,^8^,^9^,^10^. For instance, the shot noise measurements can be used to probe the dynamical in an open double quantum dots (QDs)^11^, the coherent coupling between serially coupled QDs^12^, the evolution of the Kondo effect in a QD^13^, and the conduction channels of quantum conductors^14^. In particular, shot noise characteristics can provide information about the feature of the pseudospin Kondo effect in a laterally coupled double QDs^15^, the spin accumulations in a electron reservoir^16^, and the charge fractionalization in the *ν* = 2 quantum Hall edge^17^. In addition, the degree of entanglement of two electrons in the double QDs^18^, the dephasing rate in a closed QD^19^, the internal level structure of single molecule magnet^20^,^21^ can be characterized by the super-Poissonian shot noise.

On the other hand, the quantum coherence in coupled QD system, which is characterized by the off-diagonal elements of the reduced density matrix of the QD system within the framework of the density matrix theory^22^, plays an important role in the electron tunneling processes and has a significant influence on electron transport^23^,^24^,^25^,^26^,^27^,^28^,^29^,^30^,^31^,^32^,^33^. In particular, theoretical studies have demonstrated that the high-order cumulants, e.g., the shot noise, the skewness, are more sensitive to the quantum coherence than the average current in the different types of QD systems^12^,^34^,^35^,^36^,^37^,^38^ and the quantum coherence information in a side-coupled double QD system can be extracted from the high-order current cumulants^35^. In fact, the non-Markovian dynamics of the QD system also plays an important role in the non-equilibrium electron tunneling processes. However, the above studies on current noise or FCS were mainly based on the different types of Markovian master equations. Although the influence of non-Markovian effect on the long-time limit of the FCS in the QD systems has received some attention^33^,^39^,^40^,^41^,^42^,^43^,^44^,^45^,^46^, how the non-Markovian effect affects the FCS is still an open issue, especially the influence of the interplay between the quantum coherence and non-Markovian effect on the long-time limit of the FCS has not yet been revealed.

The aim of this report is thus to derive a non-Markovian FCS formalism based on the exact time-convolutionless (TCL) master equation and study the influences of the quantum coherence and non-Markovian effect on the FCS in QD molecule systems. It is demonstrated that the non-Markovian effect manifests itself through the quantum coherence of the considered QD molecule system, and has a significant impact on the FCS in the high quantum-coherent QD molecule system, which depends on the coupling of the considered QD molecule system with the incident and outgoing electrodes. Consequently, it is necessary to consider the influence of the non-Markovian effect on the full counting statistics of electron transport in a high quantum-coherent single-molecule system.

## Results

**Figure 1:**
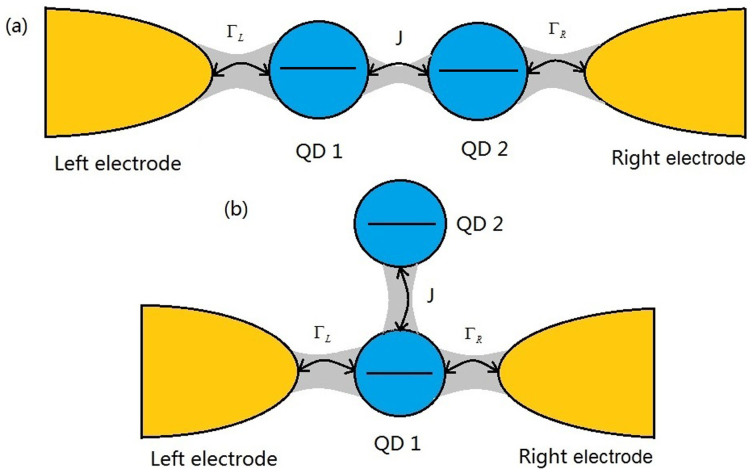
Schematic of the two single-level QD molecules weakly coupled to two electrodes, (a) serially coupled double QDs, (b) side-coupled double QDs. Here, the two QD molecules possess high quantum coherence in the case of 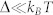 (Δ being the singly-occupied eigenenergy separation, *k_B_* the Boltzmann constant, *T* the temperature of the QDs system). The hopping coupling between the two QDs, and the strength of coupling between the QDs system and the electrode *α*, are characterized by *J* and Γ*_α_*, respectively.

We now study the influences of the quantum coherence and non-Markovian effect on the FCS of electronic transport through the QD molecule system. In order to facilitate discussions effectively, we consider three typical QD systems, namely, single QD without quantum coherence, serially coupled double QDs and side-coupled double QDs with high quantum coherence in a certain parameter regime (see Fig. 1). In addition, we assume the bias voltage (*μ_L_* = −*μ_R_* = *V_b_*/2) is symmetrically entirely dropped at the QD-electrode tunnel junctions, which implies that the levels of the QDs are independent of the applied bias voltage even if the couplings are not symmetric, and choose meV as the unit of energy which corresponds to a typical experimental situation^47^.

### Single quantum dot without quantum coherence

In this subsection, we consider a single QD weakly coupled to two ferromagnetic electrodes. The Hamiltonian of the considered system is described by the *H_total_* = *H_dot_* + *H_leads_* + *H_T_*. The QD Hamiltonian *H_dot_* is given by
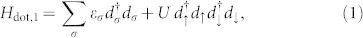
where 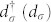 creates (annihilates) an electron with spin *σ* and on-site energy *ε_σ_* (which can be tuned by a gate voltage *V_g_*) in this QD system. *U* is the intradot Coulomb interaction between two electrons in the QD system.

The relaxation in the two ferromagnetic electrodes is assumed to be sufficiently fast, so that their electron distributions can be described by equilibrium Fermi functions. The two electrodes are thus modeled as non-interacting Fermi gases and the corresponding Hamiltonians can be expressed as
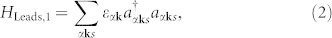
where 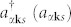 creates (annihilates) an electron with energy *ε_α_*_**k**_, spin *s* and momentum **k** in *α* (*α* = *L*, *R*) electrode, and *s* = + (−) denotes the majority (minority) spin states with the density of states *g_α_*_,*s*_. The polarization vectors **p***_L_* (left lead) and **p***_R_* (right lead) are parallel to each other, and their magnitudes are characterized by *p_α_* = |**p***_α_*| = (*g_α_*_,+_ − *g_α_*_,−_)/(*g_α_*_,+_ + *g_α_*_,−_). The tunneling between the QD and the electrodes is described by
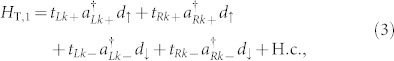
where spin-up ↑ and spin-down ↓ are defined to be the majority spin and minority spin of the ferromagnet, respectively.

The QD-electrode coupling is assumed to be sufficiently weak, thus, the sequential tunneling is dominant and can be well described by the quantum master equation of reduced density matrix spanned by the eigenstates of the QD. The particle-number-resolved TCL quantum master equation for the reduced density matrix of the considered single QD is given by
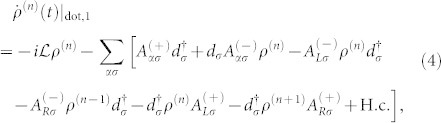
For more details, see Methods section. Here, the complete basis {|0, 0〉, |↑, 0〉, |↓, 0〉, |↑, ↓〉} is chosen to describe the electronic states of this single QD system, and the single QD system parameters are chosen as 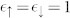, *U* = 5, *p* = 0.9 and *k_B_T* = 0.04.

**Figure 2:**
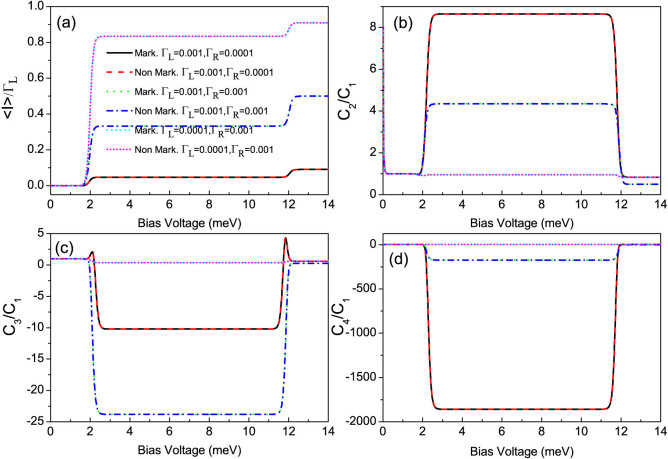
The average current (〈*I*〉), shot noise (*C*_2_/*C*_1_), skewness (*C*_3_/*C*_1_) and kurtosis (*C*_4_/*C*_1_) versus bias voltage for the Morkovian and the non-Markovian case at different coupling of the single QD with two ferromagnetic electrodes, respectively. Here, *C_k_* is the zero-frequency *k*-order cumulant of current fluctuations. The non-Markovian effect has no influence on the first four current cumulants of the considered single QD. The single QD system parameters: 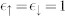, *U* = 5, *p* = 0.9 and *k_B_T* = 0.04.

Figure 2 shows the first four current cumulants as a function of the bias voltage for different ratios Γ*_L_*/Γ*_R_* describing the left-right asymmetry of the QD-electrode coupling. We found that the non-Markovian effect has no influence on the current noise behaviors of the single QD considered here, see Fig. 2. Scrutinizing Eq. (4), it is found that for the non-Markovian case the elements of the reduced density matrix are equivalent to that for the Markovian case because there are not the off-diagonal elements of the reduced density matrix. Thus, the equations of motion of the four elements of the reduced density matrix can be expressed as
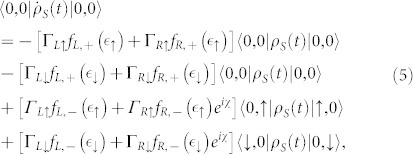

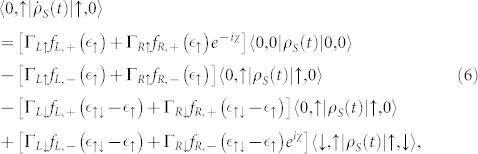

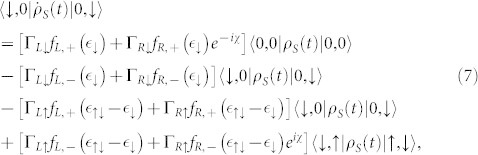

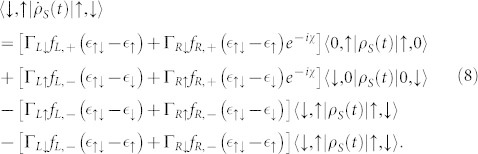
Here, *f_α_*_,+_ is the Fermi function of the electrode *α*, and *f_α_*_,−_ = 1−*f_α_*_,+_. The detailed procedure for calculation of the equation of motion of a reduced density matrix, see Methods section. Within the framework of the density matrix theory, the off-diagonal elements of the reduced density matrix characterize the quantum coherence of the considered QD system. Thus, the influence of the non-Markovian effect on the FCS may be associated with the quantum coherence of the considered QD system. In order to confirm this conclusion, we take serially coupled and side-coupled double QDs for illustration in the following two subsection.

### Serially coupled double quantum dots with high quantum coherence

We now consider two serially coupled double QDs weakly connected to two metallic electrodes, see Fig. 1(a). For the sake of simplicity, the spin degree of freedom has not been considered. The double-QD is described by a spinless Hamiltonian
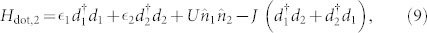
where 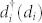 creates (annihilates) an electron with energy *ε_i_* (which can be tuned by a gate voltage *V_g_*) in *i*th QD. *U* is the interdot Coulomb repulsion between two electrons in the double QD system, where we consider the intradot Coulomb interaction *U* → ∞, so that the double-electron occupation in the same QD is prohibited. The last term of *H_dot_* describes the hopping coupling between the two dots with *J* being the hopping parameter. The two metallic electrodes are modeled as non-interacting Fermi gases and the corresponding Hamiltonians are given by
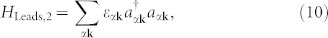
where 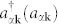 creates (annihilates) an electron with energy *ε_α_*_**k**_ and momentum **k** in *α* (*α* = *L*, *R*) electrode. The tunneling between the double QDs and the two electrodes is described by
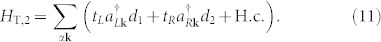
For the case of the weak QD-electrode coupling, the particle-number-resolved TCL quantum master equation for the reduced density matrix of the considered serially double-QD system reads
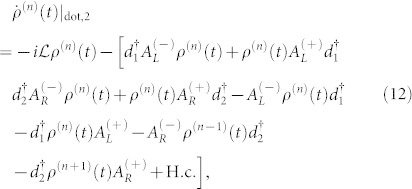
Here, we can diagonalize the serially coupled double QDs Hamiltonian *H*_dot,2_ in the basis represented by the electron occupation numbers in the QD-1 and QD-2 denoted respectively by *N_L_* and *N_R_*, namely, {|0, 0〉, |1, 0〉, |0, 1〉, |1, 1〉}, and obtain the corresponding four eigenstates of the considered serially coupled double QDs system^48^
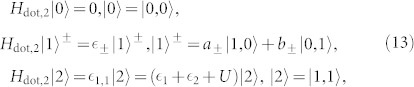
with
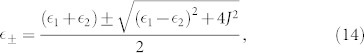
and
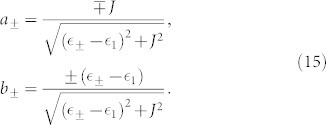
Here, we focus on the regime 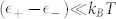, where the hopping coupling between the two QDs strongly modifies the internal dynamics, and the off-diagonal elements of the reduced density matrix play an essential role in the electron tunneling processes^23^,^49^,^50^,^51^. In the following numerical calculations, thus, the parameters of the serially coupled double QDs system are chosen as 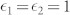, *J* = 0.001, *U* = 4 and *k_B_T* = 0.05.

**Figure 3:**
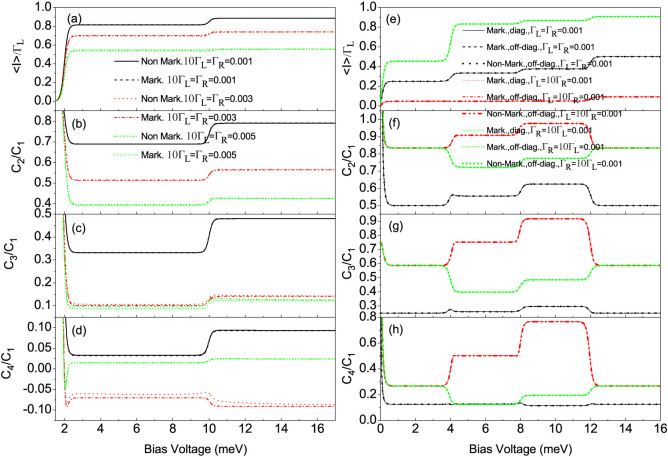
(a)–(d) The average current (〈*I*〉), shot noise (*C*_2_/*C*_1_), skewness (*C*_3_/*C*_1_) and kurtosis (*C*_4_/*C*_1_) versus bias voltage for the Morkovian and the non-Markovian case at different values of the QD-2-electrode coupling Γ*_R_* with Γ*_L_*/Γ*_R_* = 0.1. Here, *C_k_* is the zero-frequency *k*-order cumulant of current fluctuations. The non-Markovian effect in the Γ*_L_*/Γ*_R_* = 0.1 case has a weak influence on the the first four current cumulants. The serially coupled double QDs system parameters: 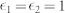, *J* = 0.001, *U* = 4 and *k_B_T* = 0.05. (e)–(h) The average current (〈*I*〉), shot noise (*C*_2_/*C*_1_), skewness (*C*_3_/*C*_1_) and kurtosis (*C*_4_/*C*_1_) versus bias voltage for different coupling of the serially coupled double QDs system with two metallic electrodes. Here three cases are considered, namely, (1) the Markovian and the diagonal elements of the reduced density matrix, (2) the Markovian and the off-diagonal elements of the reduced density matrix, (3) the non-Markovian and the off-diagonal elements of the reduced density matrix. The non-Markovian effect has a very weak influence on the first four current cumulants in the serially coupled double QD system with a relatively small quantum coherence. The serially coupled double QDs system parameters: 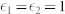, *J* = 1, *U* = 4 and *k_B_T* = 0.05.

**Figure 4:**
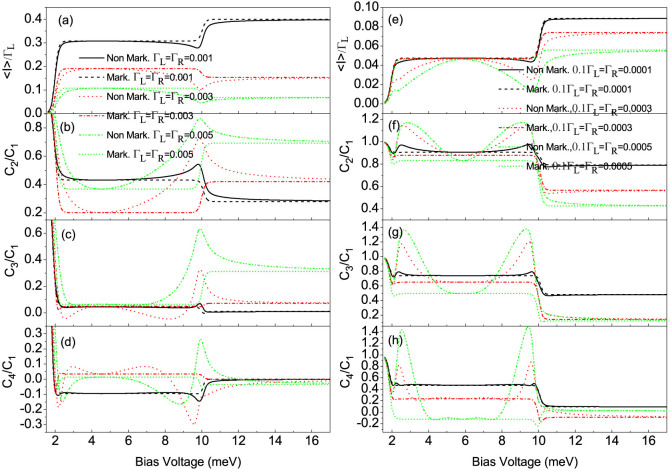
The average current (〈*I*〉), shot noise (*C*_2_/*C*_1_), skewness (*C*_3_/*C*_1_) and kurtosis (*C*_4_/*C*_1_) versus bias voltage for the Morkovian and the non-Markovian case at different values of the QD-2-electrode coupling Γ*_R_*. (a)–(d) for Γ*_L_*_/_Γ*_R_* = 1, (e)–(h) for Γ*_L_*/Γ*_R_* = 10. Here, *C_k_* is the zero-frequency *k*-order cumulant of current fluctuations. The non-Markovian effect in the Γ*_L_*/Γ*_R_* ≥ 1 case has a significant impact on the first four cumulants of transport current. The serially coupled double QDs system parameters: 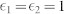, *J* = 0.001, *U* = 4 and *k_B_T* = 0.05.

When the coupling of the QD-2 with the right (drain) electrode is stronger than that of the QD-1 with the left (source) electrode, namely, Γ*_L_*/Γ*_R_* < 1, we plot the first four current cumulants as a function of the bias voltage for different values of the QD-2-electrode coupling Γ*_R_* at Γ*_L_*/Γ*_R_* = 0.1 in Figs. 3(a)–3(d). We found that the non-Markovian effect has a very weak influence on the FCS. Interestingly, the high-order current cumulants the skewness and the kurtosis can still show the tiny differences, see Figs. 3(c) and 3(d). Whereas for the Γ*_L_*/Γ*_R_* ≥ 1 case, the non-Markovian effect has a significant impact on the FCS, see Fig. 4. Especially, for a relatively large value of the ratio Γ*_L_*/Γ*_R_* = 10 and the coupling of the QD-1 with the left electrode being stronger than the hoping coupling, namely, Γ*_L_*/*J* > 1, the non-Markovian effect can induce a strong negative differential conductance (NDC) and super-Poissonian noise, see Figs. 4(e) and 4(f). In addition, in the case of Γ*_L_*/Γ*_R_* ≥ 1 and Γ*_L_*/*J* > 1, the transitions of the skewness and the kurtosis from positive (negative) to negative (positive) values are observed, see the dotted line in Fig. 4(c), the dotted and dash-dot-dotted lines in Fig. 4(d), and the dash-dot-dotted line in Fig. 4(h). It is well known that the skewness and the kurtosis (both its magnitude and sign) characterize, respectively, the asymmetry of and the peakedness of the distribution around the average transferred-electron number 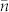 during a time interval *t*, thus that provides further information for the counting statistics beyond the shot noise.

**Figure 5:**
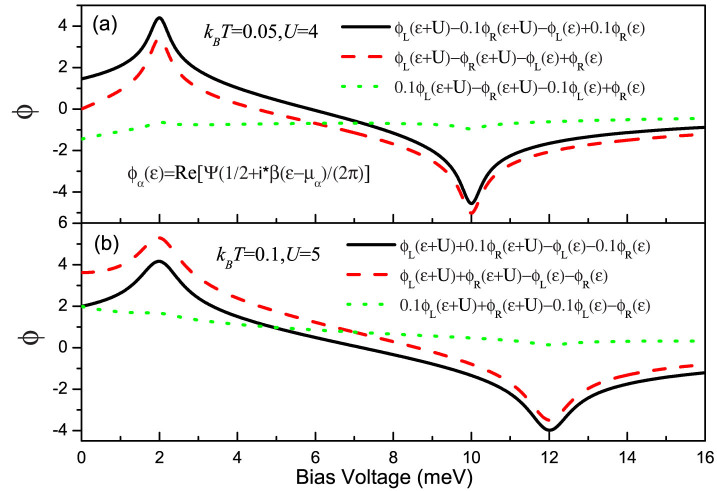
(a) The functions Φ*_L_* − 0.1Φ*_R_* (Γ*_R_* = 0.1Γ*_L_*), Φ*_L_* − Φ*_R_* (Γ*_R_* = Γ*_L_*) and 0.1Φ*_L_* − Φ*_R_* (Γ*_L_* = 0.1Γ*_R_*) as a function of bias voltage with *U* = 4 and *k_B_T* = 0.05. (b) The functions Φ*_L_* + 0.1Φ*_R_* (Γ*_R_* = 0.1Γ*_L_*), Φ*_L_* + Φ*_R_* (Γ*_R_* = Γ*_L_*) and 0.1Φ*_L_* + Φ*_R_* (Γ*_L_* = 0.1Φ*_R_*) as a function of bias voltage with *U* = 5 and *k_B_T* = 0.1. Here, 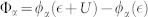, 
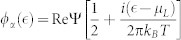
 and Ψ is the digamma function. The variation of the value of the above mentioned function is responsible for whether the non-Markovian effect has a remarkable influence on the first four cumulants of transport current.

To discuss the underlying mechanisms of the current noise clearly, for the system parameters considered here, the two singly-occupied eigenstates and eigenvalues can be expressed as
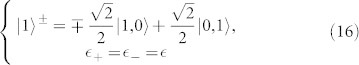
Here we have utilized the equations 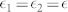 and 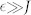. In this situation, the equations of motion of the six elements of the reduced density matrix are given by
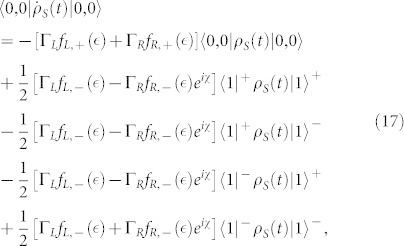

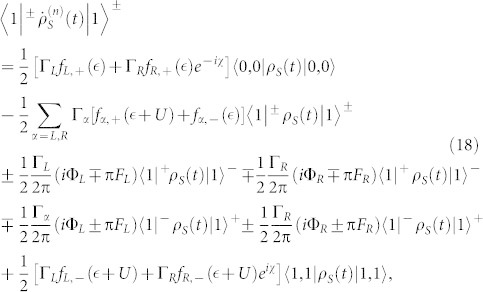

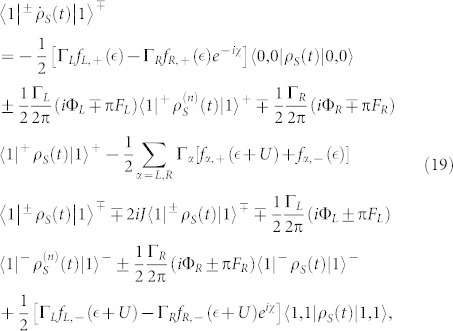

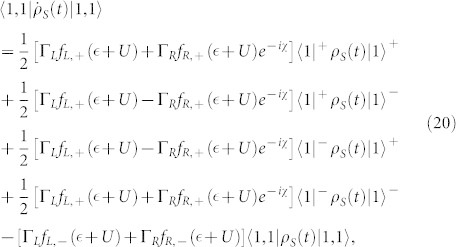
where 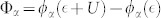, 
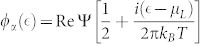
 (Ψ is the digamma function) and 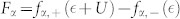. Compared with the Markovian case, it is obvious that the non-Markovian effect manifests itself through the off-diagonal elements of the reduced density matrix, namely, the quantum coherence of the considered QDs system. In Fig. 5(a), we plot the functions Φ*_L_* − 0.1Φ*_R_* (Γ*_R_* = 0.1Γ*_L_*), Φ*_L_* − Φ*_R_* (Γ*_R_* = Γ*_L_*) and 0.1Φ*_L_* − Φ*_R_* (Γ*_L_* = 0.1Γ*_R_*) as a function of bias voltage. It is clearly evident that the values of the functions Φ*_L_* − 0.1Φ*_R_* and Φ*_L_* − Φ*_R_* show significant variations with increasing bias voltage, especially in the vicinity of the bias voltages *V_b_* = 2 and *V_b_* = 10 because the new transport channels begin to participate in quantum transport; while 0.1Φ*_L_* − Φ*_R_* has a gentle variation. Consequently, the non-Markovian effects in the Γ*_L_*/Γ*_R_* ≥ 1 case have a remarkable impact on the FCS, see Fig. 4. Moreover, for Γ*_L_*/Γ*_R_* = 10 case, the non-Markovian effect has a more significant on the FCS than the Γ*_L_*/Γ*_R_* = 1 case, which originates from the QD-2-electrode coupling Γ*_R_* is weaker than the hoping coupling *J*, where the electron tunneling from QD-1 can not tunnel out QD-2 very quickly and still influence the internal dynamics.

In order to illustrate whether the non-Markovian effect has a weak influence on the FCS in a relatively small quantum-coherent QD system, we consider the regime 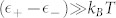 (*J* = 1), where the off-diagonal elements of the reduced density matrix have little influence on the electron tunneling processes. We find that for the *J* = 1 case the diagonal elements of the reduced density matrix play a major role in the electron tunneling processes, and the non-Markovian effect in this case indeed has little impact on the FCS, see Figs. 3(e)–3(h). Consequently, the influence of the non-Markovian effect on the FCS depends on the quantum coherence of the considered QD system. To prove whether this conclusion is universal or not, we take side-coupled double QDs for further illustration in the following subsection.

### Side-coupled double quantum dots with high quantum coherence

We consider here a side-coupled double QDs system. In this case, the QD-1 is only weakly coupled to the two electrodes, see Fig. 1(b). The QD-electrode tunneling is thus described by
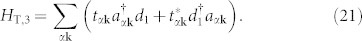


In the case of the QD-electrode weak coupling, the particle-number-resolved TCL quantum master equation for the side-coupled double QDs can be expressed as
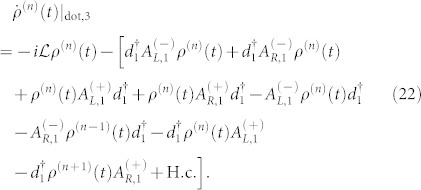
Here, the eigenstates and eigenvalues of the side-coupled double QDs system are the same as the serially coupled double QDs system. In the following numerical calculations, the parameters of the side-coupled QDs system are chosen as 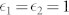, *J* = 0.001, *U* = 5 and *k_B_T* = 0.1.

**Figure 6:**
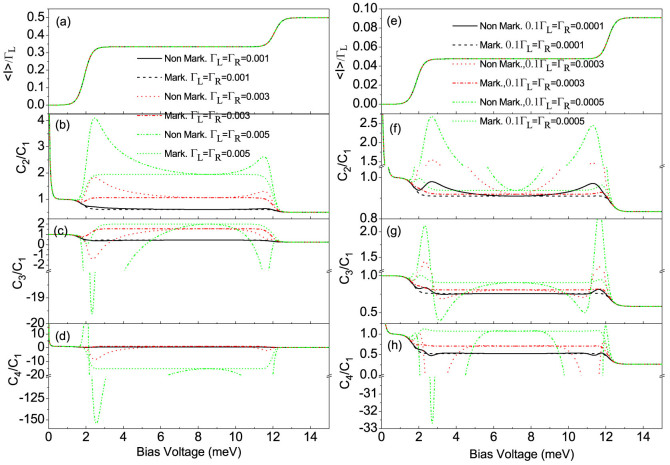
The average current (〈*I*〉), shot noise (*C*_2_/*C*_1_), skewness (*C*_3_/*C*_1_) and kurtosis (*C*_4_/*C*_1_) versus bias voltage for the Morkovian and the non-Markovian case at different values of the QD-1-electrode coupling Γ*_R_*. (a)–(d) for Γ*_L_*/Γ*_R_* = 1, (e)–(h) for Γ*_L_*/Γ*_R_* = 10. Here, *C_k_* is the zero-frequency *k*-order cumulant of current fluctuations. The non-Markovian effect in the Γ*_L_*/Γ*_R_* ≥ 1 case has a more remarkable impact on the first four cumulants of transport current than that in the serially coupled double QDs system, but the NDC does not appear. The side-coupled double QDs system parameters: 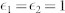, *J* = 0.001, *U* = 5 and *k_B_T* = 0.1.

**Figure 7:**
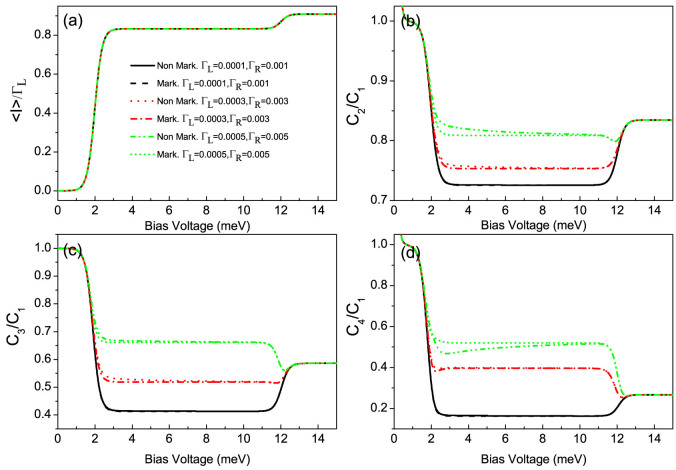
The average current (〈*I*〉), shot noise (*C*_2_/*C*_1_), skewness (*C*_3_/*C*_1_) and kurtosis (*C*_4_/*C*_1_) versus bias voltage for the Morkovian and the non-Markovian case at different values of the QD-1-electrode coupling Γ*_R_* with Γ*_L_*/Γ*_R_* = 0.1. Here, *C_k_* is the zero-frequency *k*-order cumulant of current fluctuations. The non-Markovian effect in the Γ*_L_*/Γ*_R_* = 0.1 case has a slightly influence on the the first four current cumulants. The other system parameters are the same as in Fig. 6.

For the present side-coupled QDs system with high quantum coherence, we find that for Γ*_L_*/Γ*_R_* ≥ 1 case the non-Markovian effect has a more remarkable impact on the FCS than that in the serially coupled double QDs system, but the NDC does not appear, see Figs. 4 and 6. For instance, in the case of Γ*_L_*/*J* > 1 and Γ*_L_*/Γ*_R_* = 1, the non-Markovian effect can further enhance the super-Poissonian shot noise, see the dotted and dash-dot-dotted lines in Fig. 6(b); and the transitions of the skewness and the kurtosis from a relatively small positive to a large negative values take place, especially for a relatively large value Γ*_L_*/*J* the kurtosis can be further decreased to a very large negative value, see the dotted and dash-dot-dotted lines in Figs. 6(c) and 6(d). While for the Γ*_L_*/*J* > 1 and Γ*_L_*/Γ*_R_* = 10 case the non-Markovian effect can enhance the shot noise to a super-Poissonian value, see the dotted and dash-dot-dotted lines in Fig. 6(f), and the transition of the kurtosis from small positive to large negative values only takes place, see the dotted and dash-dot-dotted lines in Fig. 6(h). For the system parameters considered here, namely, in the limit of 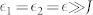, the equations of motion of the six elements of the reduced density matrix read
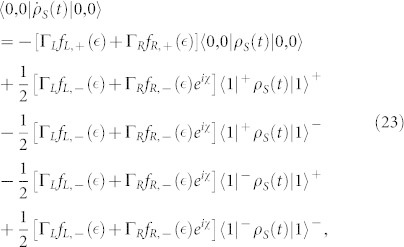

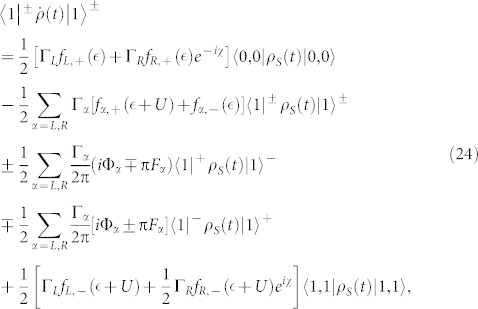

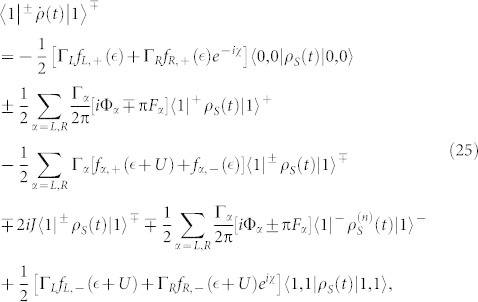

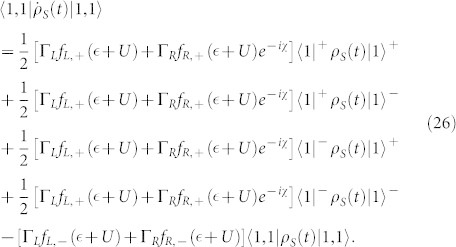
From the above four equations, we find that these characteristics also originate from the quantum coherence of the side-coupled double QDs, and can also be understood in terms of the functions Φ*_L_* + 0.1Φ*_R_* and Φ*_L_* + Φ*_R_*, which have considerable variations in the vicinity of the bias voltages *V_b_* = 2 and *V_b_* = 12 because the new transport channels begin to enter the bias voltage window, see the solid and dashed lines in Fig. 5(b). As for the Γ*_L_*/Γ*_R_* < 1 case the non-Markovian effect has a slightly influence on the FCS because the function 0.1Φ*_L_* + Φ*_R_* has a gentle variation with increasing the bias voltage, see the dotted line in Fig. 5(b), which is the same as the serially coupled double QDs system, see Figs. 3(a)–3(d) and 7.

In addition, it should be pointed out that for Γ*_L_*/Γ*_R_* = 1 the non-Markovian effect has a stronger impact on the FCS than that for Γ*_L_*/Γ*_R_* > 1 case, which is contrary to the case of the serially coupled double QDs system. For the the side-coupled double QDs system, the quantum coherence originates from the quantum interference between the direct electron tunneling process, namely, the conduction-electron tunneling into the QD-1 and then directly tunneling out of the QD-1 onto the drain electrode, and the indirect tunneling process, namely, the conduction-electron from the source electrode first tunneling from the QD-1 to the QD-2, then tunneling back into the QD-1, and at last tunneling out of the QD-1. Thus, the fast direct tunneling process in the Γ*_L_* = 10Γ*_R_* case can be suppressed compared with the Γ*_L_* = Γ*_R_* case, which leads to the non-Markovian effect has a relatively strong impact on the FCS in the Γ*_L_*/Γ*_R_* = 1 case.

## Discussion

We have developed a non-Markovian FCS formalism based on the exact TCL master equation, and studied the influence of the interplay between the quantum coherence and non-Markovian effect on the long-time limit of the FCS in three QD systems, namely, single QD, serially coupled double QDs and side-coupled double QDs. It is demonstrated that the non-Markovian effect manifests itself through the quantum coherence of the considered QD molecule system, and especially has a significant impact on the FCS in the high quantum-coherent QD molecule system, which depends on the coupling of the considered QD molecule system with the source and drain electrodes. For the single QD system without quantum coherence, the non-Markovian effect has no influence on the current noise properties; whereas for the serially coupled and side-coupled double QDs systems with high quantum coherence, that has a remarkable impact on the FCS when the coupling of the considered QD molecule with the incident electrode is equal to or stronger than that with the outgoing electrode. For instance, for the high quantum-coherent serially coupled double QDs system, the non-Markovian effect can induce a strong NDC and change the shot noise from the sub-Poissonian to super-Poissonian distribution in the case of 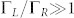 and Γ*_L_* > *J*; while for the high quantum-coherent side-coupled double QDs system, that can remarkably enhance the super-Poissonian noise or the sub-Poissonian noise for the Γ*_L_*/Γ*_R_* ≥ 1 case. Moreover, the non-Markovian effect can also lead to the occurrences of the skewness and kurtosis from small positive to large negative values. These results indicated that the influence of the non-Markovian effect on the long-time limit of the FCS should be considered in a highly quantum-coherent single-molecule system.

## Methods

### Particle-number-resolved time-convolutionless quantum master equation

We consider a general transport setup consisting of a single-level QD molecule weakly coupled to the two electrodes, see Fig. 1, which is described by the following Hamiltonian
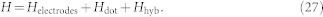
Here, the first term 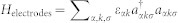 stands for the Hamiltonians of the two electrodes, with *ε_αk_* being the energy dispersion, and 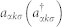 the annihilation (creation) operators in the *α* electrode. The second term 
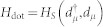
, which may contain vibrational or spin degrees of freedom and different types of many-body interaction, represents the QD molecule Hamiltonian, where 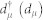 is the creation (annihilation) operator of electrons in a quantum state denoted by *μ*. The third term 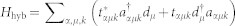 describes the tunneling coupling between the QD molecule and the two electrodes, which is assumed to be a sum of bilinear terms that each create an electron in the QD molecule and annihilate one in the electrodes or vice versa.

The QD-electrode coupling is assumed to be sufficiently weak, so that *H*_hyb_ can be treated perturbatively. In the interaction representation, the equation of motion for the total density matrix reads
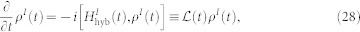
with
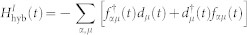
where 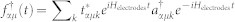 and 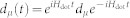. In order to derive an exact equation of motion for the reduced density matrix *ρ_S_* of the QD molecule system, it is convenient to define a super-operator 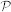 according to
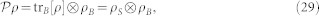
with *ρ_B_* being some fixed state of the electron electrode. Accordingly, a complementary super-operator 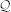 reads
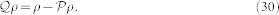
For a factorizing initial condition 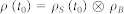, 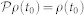, and 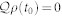. Using the TCL projection operator method^52^, one can obtain the second-order TCL master equation
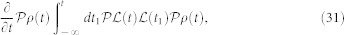
The Eq. (31) is the starting point of deriving the particle-number-resolved quantum master equation. Using Eqs. (28) and (29), after some algebraic calculations we can rewrite Eq. (31) as
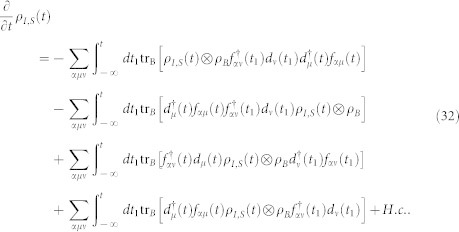


In order to fully describe the electron transport problem, we should record the number of electrons arriving at the drain electrode, which emitted from the source electrode and passing through the QD molecule. We follow Li and co-authors^53^,^54^ and introduce the Hilbert subspace *B*^(*n*)^ (*n* = 1, 2, …) corresponding to *n* electrons arriving at the drain electrode, which is spanned by the product of all many-particle states of the two isolated electrodes, and formally denoted as 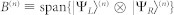. Then, the entire Hilbert space of the two electrodes can be expressed as 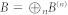. With this classification of the electrode states, the average over states in the entire Hilbert space *B* in Eq. (32) should be replaced with the states in the subspace *B*^(*n*)^, and leading to a conditional TCL master equation
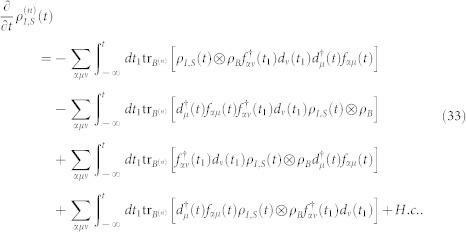


To proceed, two physical considerations are further implemented. (i) Instead of the conventional Born approximation for the entire density matrix 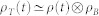, the ansatz 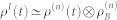 is proposed, where 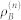 being the electrode density operator associated with *n* electrons arriving at the drain electrode. With this ansatz for the entire density operator, tracing over the subspace *B*^(*n*)^, the Eq. (33) can be rewritten as
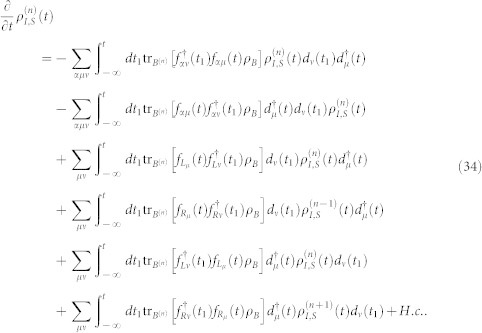
Here we have used the orthogonality between the states in different subspaces. (ii) The extra electrons arriving at the drain electrode will flow back into the source electrode via the external closed transport circuit. Moreover, the rapid relaxation processes in the electrodes will bring the electrodes to the local thermal equilibrium states quickly, which are determined by the chemical potentials. Consequently, after the procedure done in Eq. (34), the electrode density matrices 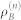 and 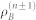 should be replaced by 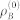. In the Schrödinger representation, the Eq. (34) can be expressed as
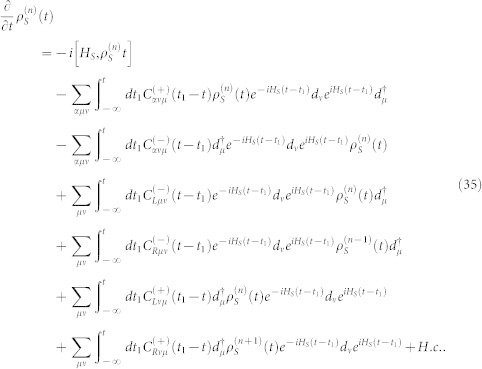
where the correlation function are defined as
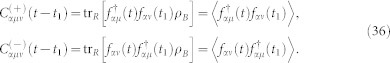
Introducing the following super-operators
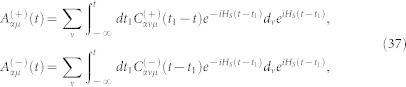
then, the Eq. (35) can be rewritten as a compact form
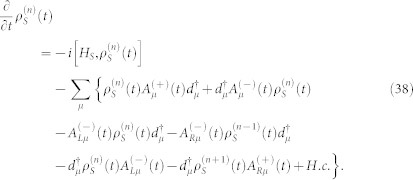
where 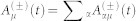. The above equation is the starting point of the non-Markovian FCS calculation.

### Non-Markovian full counting statistics

In this subsection, we outline the procedure to calculate the non-Markovian FCS based on Eq. (38). The FCS can be obtained from the cumulant generating function (CGF) *F* (*χ*) which related to the probability distribution *P* (*n*, *t*) by^54^,^55^ 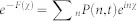, where *χ* is the counting field. The CGF *F* (*χ*) connects with the particle-number-resolved density matrix *ρ*^(*n*)^ (*t*) by defining 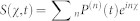. Evidently, we have *e*^−*F*(*χ*)^ = Tr[*S* (*χ*, *t*)], where the trace is over the eigenstates of the QD molecule system. Since Eq. (38) has the following form 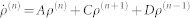, then, *S* (*χ*, *t*) satisfies 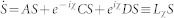, where *S* is a column matrix, and *A*, *C* and *D* are three square matrices. The specific form of *L_χ_* can be obtained by performing a discrete Fourier transformation to the matrix element of Eq. (38). In the low frequency limit, the counting time, namely, the time of measurement is much longer than the time of tunneling through the QD molecule system. In this case, *F* (*χ*) is given by^34^,^40^,^43^,^55^,^56^,^57^ *F* (*χ*) = −*λ*_1_ (*χ*) *t*, where *λ*_1_ (*χ*) is the eigenvalue of *L_χ_* which goes to zero for *χ* → 0. According to the definition of the cumulants one can express *λ*_1_ (*χ*) as 
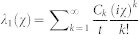
. The low order cumulants can be calculated by the Rayleigh–Schrödinger perturbation theory in the counting parameter *χ*. In order to calculate the first four current cumulants we expand *L_χ_* to four order in *χ*
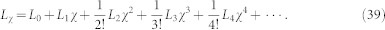
and define the two projectors^40^,^43^,^56^,^58^ 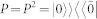 and *Q* = *Q*^2^ = 1 − *P*, obeying the relations *PL*_0_ = *L*_0_*P* = 0 and *QL*_0_ = *L*_0_*Q* = *L*_0_. Here, |0〉〉 is the right eigenvector of *L*_0_, i.e., *L*_0_ |0〉〉 = 0, and 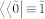 is the corresponding left eigenvector. In view of *L*_0_ being singular, we also introduce the pseudoinverse according to 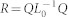, which is well-defined due to the inversion being performed only in the subspace spanned by *Q*. After a careful calculation, *λ*_1_ (*χ*) is given by
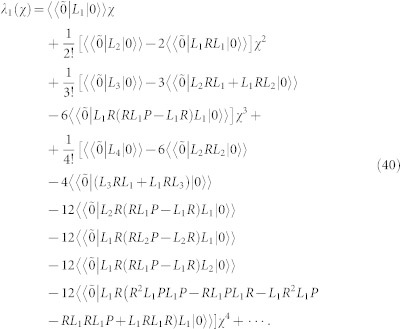
From Eq. (40) we can identify the first four current cumulants:
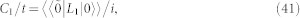

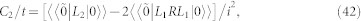

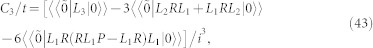

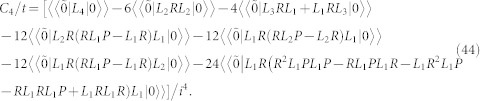
Here, it is important to emphasize that the first four cumulants *C_k_* are directly related to the transport characteristics. For example, the first-order cumulant (the peak position of the distribution of transferred-electron number) 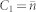 gives the average current 〈*I*〉 = *eC*_1_/*t*. The zero-frequency shot noise is related to the second-order cumulant (the peak-width of the distribution) 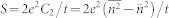. The third-order cumulant 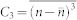 and four-order cumulant 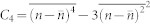 characterize, respectively, the skewness and kurtosis of the distribution. Here, 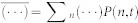. In general, the shot noise, skewness and kurtosis are represented by the Fano factor *F*_2_ = *C*_2_/*C*_1_, *F*_3_ = *C*_3_/*C*_1_ and *F*_4_ = *C*_4_/*C*_1_, respectively.

## Author Contributions

H.B.X. conceived the idea and designed the research and performed calculations. H.J.J., J.Q.L. and W.M.L. contributed to the analysis and interpretation of the results and prepared the manuscript.

## References

[b1] Levitov L. S., Lee H. & Lesovik G. B. Electron counting statistics and coherent states of electric current. J. Math. Phys. 37, 4845 (1996).

[b2] Blanter Y. M. & Büttiker M. Shot noise in mesoscopic conductors. Phys. Rep. 336, 1–166 (2000).

[b3] Nazarov Y. V. Quantum Noise in Mesoscopic Physics (edited by Kluwer, Dordrecht, 2003).

[b4] Gustavsson S. *et al.* Counting Statistics of Single Electron Transport in a Quantum Dot. Phys. Rev. Lett. 96, 076605 (2006). 16606120 10.1103/PhysRevLett.96.076605

[b5] Fujisawa T., Hayashi T., Tomita R. & Hirayama Y. Bidirectional Counting of Single Electrons. Science 312, 1634 (2006). 16778051 10.1126/science.1126788

[b6] Flindt C. *et al.* Universal oscillations in counting statistics. Proc. Natl. Acad. Sci. U.S.A. 106, 10116 (2009). 19515823 10.1073/pnas.0901002106 PMC2700917

[b7] Fricke C., Hohls F., Sethubalasubramanian N., Fricke L. & Haug R. J. High-order cumulants in the counting statistics of asymmetric quantum dots. Appl. Phys. Lett. 96, 202103 (2010).

[b8] Ubbelohde N., Fricke C., Flindt C., Hohls F. & Haug R. J. Measurement of finite-frequency current statistics in a single-electron transistor. Nat. Comms. 3, 612 (2012). 10.1038/ncomms1620 PMC3272564 22215087

[b9] Fricke L. *et al.* Counting Statistics for Electron Capture in a Dynamic Quantum Dot. Phys. Rev. Lett. 110, 126803 (2013). 25166833 10.1103/PhysRevLett.110.126803

[b10] Maisi V. F., Kambly D., Flindt C. & Pekola J. P. Full Counting Statistics of Andreev Tunneling. Phys. Rev. Lett. 112, 036801 (2014). 24484157 10.1103/PhysRevLett.112.036801

[b11] Aguado R. & Brandes T. Shot Noise Spectrum of Open Dissipative Quantum Two-Level Systems. Phys. Rev. Lett. 92, 206601 (2004). 15169372 10.1103/PhysRevLett.92.206601

[b12] Kießlich G., Schöll E., Brandes T., Hohls F. & Haug R. J. Noise Enhancement due to Quantum Coherence in Coupled Quantum Dots. Phys. Rev. Lett. 99, 206602 (2007). 18233172 10.1103/PhysRevLett.99.206602

[b13] Yamauchi Y. *et al.* Evolution of the Kondo Effect in a Quantum Dot Probed by Shot Noise. Phys. Rev. Lett. 106, 176601 (2011). 21635054 10.1103/PhysRevLett.106.176601

[b14] Vardimon R., Klionsky M. & Tal O. Experimental determination of conduction channels in atomic-scale conductors based on shot noise measurements. Phys. Rev. B 88, 161404(R) (2013).

[b15] Kubo T., Tokura Y. & Tarucha S. Kondo effects and shot noise enhancement in a laterally coupled double quantum dot. Phys. Rev. B 83, 115310 (2011).

[b16] Meair J., Stano P. & Jacquod P. Measuring spin accumulations with current noise. Phys. Rev. B 84, 073302 (2011).

[b17] Milletarì M. & Rosenow B. Shot-Noise Signatures of Charge Fractionalization in the *ν* = 2 Quantum Hall Edge. Phys. Rev. Lett. 111, 136807 (2013). 24116806 10.1103/PhysRevLett.111.136807

[b18] Bodoky F., Belzig W. & Bruder C. Connection between noise and quantum correlations in a double quantum dot. Phys. Rev. B 77, 035302 (2008).

[b19] Dubrovin D. & Eisenberg E. Super-Poissonian shot noise as a measure of dephasing in closed quantum dots. Phys. Rev. B 76, 195330 (2007).

[b20] Xue H. B., Nie Y. H., Li Z. J. & Liang J. Q. Tunable electron counting statistics in a single-molecule magnet. J. Appl. Phys. 108, 033707 (2010).

[b21] Xue H. B., Nie Y. H., Li Z. J. & Liang J. Q. Effect of finite Coulomb interaction on full counting statistics of electronic transport through single-molecule magnet. Phys. Lett. A 375, 716 (2011).

[b22] Blum K. Density Matrix Theory and Applications, third ed. (Springer, Dordrecht, 2012).

[b23] Gurvitz S. A. & Prager Y. S. Microscopic derivation of rate equations for quantum transport. Phys. Rev. B 53, 15932 (1996). 10.1103/physrevb.53.15932 9983432

[b24] Braun M., König J. & Martinek J. Theory of transport through quantum-dot spin valves in the weak-coupling regime. Phys. Rev. B 70, 195345 (2004).

[b25] Wunsch B., Braun M., König J. & Pfannkuche D. Probing level renormalization by sequential transport through double quantum dots. Phys. Rev. B 72, 205319 (2005).

[b26] Djuric I., Dong B. & Cui H. L. Super-Poissonian shot noise in the resonant tunneling due to coupling with a localized level. Appl. Phys. Lett. 87, 032105(2005).

[b27] Djuric I., Dong B. & Cui H. L. Theoretical investigations for shot noise in correlated resonant tunneling through a quantum coupled system. J. Appl. Phys. 99, 063710 (2006).

[b28] Harbola U., Esposito M. & Mukamel S. Quantum master equation for electron transport through quantum dots and single molecules. Phys. Rev. B 74, 235309 (2006).

[b29] Pedersen J. N., Lassen B., Wacker A. & Hettler M. H. Coherent transport through an inter-acting double quantum dot: Beyond sequential tunneling. Phys. Rev. B 75, 235314 (2007).

[b30] Begemann G., Darau D., Donarini A. & Grifoni M. Symmetry fingerprints of a benzene single-electron transistor: Interplay between Coulomb interaction and orbital symmetry. Phys. Rev. B 77, 201406 (R) (2008).

[b31] Darau D., Begemann G., Donarini A. & Grifoni M. Interference effects on the transport characteristics of a benzene single-electron transistor. Phys. Rev. B 79, 235404 (2009).

[b32] Schultz M. G. & von Oppen F. Quantum transport through nanostructures in the singular-coupling limit. Phys. Rev. B 80, 033302 (2009).

[b33] Schaller G., Kießlich G. & Brandes T. Transport statistics of interacting double dot systems: Coherent and non-Markovian effects. Phys. Rev. B 80, 245107 (2009).

[b34] Kießlich G., Samuelsson P., Wacker A. & Schöll E. Counting statistics and decoherence in coupled quantum dots. Phys. Rev. B 73, 033312 (2006).

[b35] Xue H. B. Full counting statistics as a probe of quantum coherence in a side-coupled double quantum dot system. Annals of Physics (New York) 339, 208 (2013).

[b36] Welack S., Esposito M., Harbola U. & Mukamel S. Interference effects in the counting statistics of electron transfers through a double quantum dot. Phys. Rev. B 77, 195315 (2008).

[b37] Fang T. F., Wang S. J. & Zuo W. Flux-dependent shot noise through an Aharonov-Bohm interferometer with an embedded quantum dot. Phys. Rev. B 76, 205312 (2007).

[b38] Fang T. F., Zuo W. & Chen J. Y. Fano effect on shot noise through a Kondo-correlated quantum dot. Phys. Rev. B 77, 125136 (2008).

[b39] Braggio A., König J. & Fazio R. Full Counting Statistics in Strongly Interacting Systems: Non-Markovian Effects. Phys. Rev. Lett. 96, 026805 (2006). 16486615 10.1103/PhysRevLett.96.026805

[b40] Flindt C., Novotný T., Braggio A., Sassetti M. & Jauho A. P. Counting Statistics of Non-Markovian Quantum Stochastic Processes. Phys. Rev. Lett. 100, 150601 (2008). 18518090 10.1103/PhysRevLett.100.150601

[b41] Zedler P., Schaller G., Kiesslich G., Emary C. & Brandes T. Weak-coupling approximations in non-Markovian transport. Phys. Rev. B 80, 045309 (2009).

[b42] Emary C. Counting statistics of cotunneling electrons. Phys. Rev. B 80, 235306 (2009).

[b43] Flindt C., Novotný T., Braggio A. & Jauho A. P. Counting statistics of transport through Coulomb blockade nanostructures: High-order cumulants and non-Markovian effects. Phys. Rev. B 82, 155407 (2010).

[b44] Marcos D., Emary C., Brandes T. & Aguado R. Non-Markovian effects in the quantum noise of interacting nanostructures. Phys. Rev. B 83, 125426 (2011).

[b45] Emary C. & Aguado R. Quantum versus classical counting in non-Markovian master equations. Phys. Rev. B 84, 085425 (2011).

[b46] Zedler P., Emary C., Brandes T. & Novotný T. Noise calculations within the second-order von Neumann approach. Phys. Rev. B 84, 233303 (2011).

[b47] Elzerman J. M. *et al.* Single-shot read-out of an individual electron spin in a quantum dot. Nature 430, 431–435 (2004). 15269762 10.1038/nature02693

[b48] Xue H. B., Zhang Z. X. & Fei H. M. Tunable super-Poissonian noise and negative differential conductance in two coherent strongly coupled quantum dots. Eur. Phys. J. B 85, 336 (2012).

[b49] Stoof T. H. & Nazarov Y. V. Time-dependent resonant tunneling via two discrete states. Phys. Rev. B 53, 1050 (1996). 10.1103/physrevb.53.1050 9983552

[b50] Aguado R. & Brandes T. Shot Noise Spectrum of Open Dissipative Quantum Two-Level Systems. Phys. Rev. Lett. 92, 206601 (2004). 15169372 10.1103/PhysRevLett.92.206601

[b51] Luo J. Y. *et al.* Full counting statistics of level renormalization in electron transport through double quantum dots. J. Phys.: Condens. Matter 23, 145301 (2011). 21430309 10.1088/0953-8984/23/14/145301

[b52] Breuer H. P. & Petruccione F. The Theory of Open Quantum Systems (Oxford University Press, Oxford, 2002).

[b53] Li X. Q., Luo J., Yang Y. G., Cui P. & Yan Y. J. Quantum master-equation approach to quantum transport through mesoscopic systems. Phys. Rev. B 71, 205304 (2005).

[b54] Wang S. K., Jiao H. J., Li F., Li X. Q. & Yan Y. J. Full counting statistics of transport through two-channel Coulomb blockade systems. Phys. Rev. B 76, 125416 (2007).

[b55] Bagrets D. A. & Nazarov Y. V. Full counting statistics of charge transfer in Coulomb blockade systems. Phys. Rev. B 67, 085316 (2003).

[b56] Flindt C., Novotný T. & Jauho A. P. Full counting statistics of nano-electromechanical systems. EPL 69, 475 (2005).

[b57] Groth C. W., Michaelis B. & Beenakker C. W. J. Counting statistics of coherent population trapping in quantum dots. Phys. Rev. B 74, 125315 (2006).

[b58] Xue H. B., Nie Y. H., Li Z. J. & Liang J. Q. Effects of magnetic field and transverse anisotropy on full counting statistics in single-molecule magnet. J. Appl. Phys. 109, 083706 (2011).

